# Oral capecitabine *vs* intravenous 5-fluorouracil and leucovorin: integrated efficacy data and novel analyses from two large, randomised, phase III trials

**DOI:** 10.1038/sj.bjc.6601676

**Published:** 2004-02-24

**Authors:** E Van Cutsem, P M Hoff, P Harper, R M Bukowski, D Cunningham, P Dufour, U Graeven, J Lokich, S Madajewicz, J A Maroun, J L Marshall, E P Mitchell, G Perez-Manga, P Rougier, W Schmiegel, J Schoelmerich, A Sobrero, R L Schilsky

**Affiliations:** 1 University Hospital Gasthuisberg, Leuven, Belgium; 2 M.D. Anderson Cancer Center, Houston, TX, USA; 3 Guy's and St Thomas' Hospital, London, UK; 4 Cleveland Clinic, Cleveland, OH, USA; 5 Royal Marsden Hospital, London, UK; 6 Hospital Hautepierre, Strasbourg, France; 7 Ruhr-Universität, Bochum, Germany; 8 Cancer Center of Boston, Boston, MA, USA; 9 SUNY Health Science Center, Stonybrook, NY, USA; 10 Ottawa Regional Cancer Center, Ottawa, Canada; 11 Lombardi Cancer Center, Washington, DC, USA; 12 Thomas Jefferson University, Philadelphia, PA, USA; 13 Hospital Gregorio Marañón, Madrid, Spain; 14 Hôpital Ambroise Paré, Boulogne, France; 15 Klinikum der Universität Regensburg, Regensburg, Germany; 16 Clinica Oncologica Piazza SM Maria, Udine, Italy; 17 University of Chicago, Chicago, IL, USA

**Keywords:** capecitabine, colorectal cancer, fluoropyrimidine, efficacy, oral

## Abstract

This study evaluates the efficacy of capecitabine using data from a large, well-characterised population of patients with metastatic colorectal cancer (mCRC) treated in two identically designed phase III studies. A total of 1207 patients with previously untreated mCRC were randomised to either oral capecitabine (1250 mg m^−2^ twice daily, days 1−14 every 21 days; *n*=603) or intravenous (i.v.) bolus 5-fluorouracil/leucovorin (5-FU/LV; Mayo Clinic regimen; *n*=604). Capecitabine demonstrated a statistically significant superior response rate compared with 5-FU/LV (26 *vs* 17%; *P*<0.0002). Subgroup analysis demonstrated that capecitabine consistently resulted in superior response rates (*P*<0.05), even in patient subgroups with poor prognostic indicators. The median time to response and duration of response were similar and time to progression (TTP) was equivalent in the two arms (hazard ratio (HR) 0.997, 95% confidence interval (CI) 0.885–1.123, *P*=0.95; median 4.6 *vs* 4.7 months with capecitabine and 5-FU/LV, respectively). Multivariate Cox regression analysis identified younger age, liver metastases, multiple metastases and poor Karnofsky Performance Status as independent prognostic indicators for poor TTP. Overall survival was equivalent in the two arms (HR 0.95, 95% CI 0.84–1.06, *P*=0.48; median 12.9 *vs* 12.8 months, respectively). Capecitabine results in superior response rate, equivalent TTP and overall survival, an improved safety profile and improved convenience compared with i.v. 5-FU/LV as first-line treatment for MCRC. For patients in whom fluoropyrimidine monotherapy is indicated, capecitabine should be strongly considered. Following encouraging results from phase I and II trials, randomised trials are evaluating capecitabine in combination with irinotecan, oxaliplatin and radiotherapy. Capecitabine is a suitable replacement for i.v. 5-FU as the backbone of colorectal cancer therapy.

The fluoropyrimidine 5-fluorouracil (5-FU) has remained the most extensively used chemotherapeutic agent in the treatment of advanced colorectal cancer for more than 40 years. During this time, many 5-FU-based regimens have been developed, including 48-h infusion or continuous infusions of 5-FU that are considered by many to be the safest and most effective.

Capecitabine (Xeloda®: F Hoffmann-La Roche, Basel, Switzerland) is an oral fluoropyrimidine carbamate rationally designed to generate 5-FU preferentially in tumour tissue through exploitation of higher intratumoral concentrations of thymidine phosphorylase (Miwa *et al*, 1998; Schüller *et al*, 2000). Human pharmacokinetic studies have shown that after oral administration, capecitabine is rapidly and almost completely absorbed through the gastrointestinal wall, thus avoiding direct intestinal exposure to 5-FU (Reigner *et al*, 2001). Capecitabine is then metabolised to 5-FU via a three-step enzymatic cascade. The final stage of this conversion is mediated by thymidine phosphorylase, an enzyme present at significantly increased concentrations in a wide range of tumour types, including colorectal, breast and gastric cancers, compared with normal tissue (Miwa *et al*, 1998). The tumour-preferential activation of capecitabine reduces systemic exposure to 5-FU and potentially improves efficacy and safety (Schüller *et al*, 2000).

As an oral agent, capecitabine enables dosing that approximates to continuous infusion 5-FU with improved convenience. Conventional infused 5-FU regimens require central venous access via a Port-a-Cath®, Hickman catheter or Groshong and the use of portable pumps, causing considerable inconvenience to patients. In addition, the rate of complications such as thrombosis and infection is reported to be as high as 20–60% with chronic venous access devices (Hartkamp *et al*, 2000; Schwarz *et al*, 2000). These factors, together with the known patient preference for oral chemotherapy (Liu *et al*, 1997; Borner *et al*, 2002), generated the need for an oral agent such as capecitabine that could achieve continuous exposure to 5-FU in a similar manner to infused regimens.

A randomised, phase II trial in patients with advanced colorectal cancer demonstrated that capecitabine is well tolerated and has substantial antitumour activity (Van Cutsem *et al*, 2000). Subsequently, two multicentre, open-label, phase III studies were conducted to compare capecitabine with intravenous (i.v.) 5-FU/leucovorin (5-FU/LV; Mayo Clinic regimen, the regulatory standard at the time), as first-line treatment for metastatic colorectal cancer (mCRC)(Hoff *et al*, 2001; Van Cutsem *et al*, 2001a). One study was conducted in Europe, Australia, Israel and Asia and the other was conducted in the USA, Canada, Brazil and Mexico. Both studies used identical protocols and an integrated analysis of all data was prospectively planned. The integrated analysis has provided an opportunity to retrospectively assess the use of capecitabine in a large, well-characterised population of patients with mCRC. Safety evaluation demonstrated that capecitabine has an improved safety profile compared with 5-FU/LV, with a significantly lower incidence of diarrhoea, stomatitis, nausea and alopecia. The lower incidence of Grade 3–4 neutropaenia with capecitabine led to significantly less neutropaenic fever/sepsis and consequently fewer hospitalisations (Cassidy *et al*, 2002). The improved safety profile of capecitabine also results in better utilisation of medical resources, leading to a pharmacoeconomic advantage for capecitabine over i.v. 5-FU. This was demonstrated in an analysis of medical resource use in the European randomised trial (Twelves *et al*, 2001). The safety data from the two trials have been reviewed extensively by Cassidy *et al* (2002). This paper provides a detailed review of the efficacy analyses of the pooled data.

## PATIENTS AND METHODS

### Trial design

The two studies used identical protocols and conduct, with the primary objective of establishing that oral capecitabine achieves a response rate at least equivalent to i.v. 5-FU/LV in patients with previously untreated mCRC (*α*-level of 2.5% and an equivalence margin of 10%). Secondary objectives were to compare additional efficacy parameters, including time to disease progression (TTP), overall survival, duration of response and time to first response, as well as safety and quality of life profiles and medical resource utilisation during treatment.

All patients recruited to the trials had received no prior cytotoxic chemotherapy for metastatic disease. Adjuvant or neo-adjuvant therapy completed at least 6 months prior to enrolment was permitted.

### Treatment

Patients were randomised to receive either oral capecitabine (1250 mg m^−2^ twice daily for 14 days followed by a 7-day rest period) or 5-FU/LV administered according to the Mayo Clinic regimen (LV 20 mg m^−2^ followed by 5-FU 425 mg m^−2^, administered as an i.v. bolus on days 1–5 every 28 days) (Hoff *et al*, 2001; Van Cutsem *et al*, 2001a). The standard capecitabine dose reduction scheme, described in detail elsewhere (Cassidy *et al*, 2002), was used for management of adverse events. Patients were treated for up to 48 weeks until disease progression or unacceptable toxicity. Treatment continuation beyond 48 weeks was permitted in patients without progressive disease at the discretion of the investigator (poststudy treatment phase).

### Evaluation of efficacy

Tumour evaluations were made at baseline and then at 6-weekly intervals during study treatment, based on standard World Health Organization (1979) criteria. In addition to the investigator assessment, an Independent Review Committee (IRC), consisting of a panel of radiologists who were blinded to study treatment, clinical condition of the patient and investigator's assessment, evaluated tumour responses solely on the basis of imaging.

## RESULTS

### Patient population

In total, 1207 patients were randomised to treatment with capecitabine (603 patients) or 5-FU/LV (604 patients). All patients were included in the efficacy analysis. The demographic and baseline characteristics of patients in the two groups were well balanced (Table 1

**Table 1:** Demographics and baseline characteristics

	>**Capecitabine**	**5-FU/LV**
	**(*n*=603)**	**(*n*=604)**
Male/female (%)	60/40	61/39
Age (years): median (range)	64 (23–86)	63 (24–87)
KPS (%): mean (range)	89 (70–100)	89 (70–100)
Colon/rectal cancer (%)	70/30	71/29
		
*Tumour differentiation* (%)		
Well differentiated	11	11
Moderately differentiated	62	63
Poorly differentiated	19	17
Undetermined/unknown	8	10
		
*Number of metastatic sites* (%)		
1	25	22
2	24	21
⩾3	52	57
		
*Metastatic sites* (%)		
Liver	77	77
Lung	33	33
Lymph nodes	33	35
Peritoneum	14	14
		
Prior adjuvant chemotherapy (%)	24	26

). As expected, in both treatment groups, the majority of patients had colon rather than rectal cancer. The proportions of patients who had previously received adjuvant chemotherapy were similar in the two groups and there was no significant difference between the groups with respect to extent or sites of metastatic disease. In general, there were no differences in the distribution of prognostic factors at baseline; although significant differences in serum alkaline phosphatase concentrations favouring the 5-FU/LV group were observed in one study (Hoff *et al*, 2001), no significant differences were apparent in the integrated data (Table 2

**Table 2:** Distribution of prognostic factors in the two treatment groups (mean values±s.d.)

	**Capecitabine (*n*=603)**	**5-FU/LV (*n*=604)**	**Wilcoxon test**	
			***Z***	***P*-value**
Alkaline phosphatase^a^ (U/l)	142.9 (121.2)	135.5 (116.4)	−1.7	0.091
ASAT (IU)	21.5 (15.5)	20.7 (15.7)	−1.5	0.13
Haemoglobin (g/dl)	12.9 (1.9)	12.9 (1.9)	0.2	0.87

a Normalised serum alkaline phosphatase.

ASAT=aspartate aminotransferase.

).

The median duration of treatment was 4.5 months in the capecitabine group and 4.6 months in the 5-FU/LV group. Safety data, including data on treatment interruption and dose modification, are described in detail elsewhere (Cassidy *et al*, 2002).

### Tumour responses

Results from the integrated analysis demonstrate a significantly superior overall response rate with capecitabine compared with 5-FU/LV (26 *vs* 17%, *P*<0.0002; Table 3

**Table 3:** Investigator-assessed tumour response rates

	**Capecitabine**	**5-FU/LV**	
	**(*n*=603)**	**(*n*=604)**	***P*-value**
*PR*+*CR* (%)	25.7	16.7	<0.0002
95% CI	22.4–29.6	13.8–19.9	
*Stable disease* (%)	48.3	52.2	
95% CI	43.7−51.8	48.1−56.2	

**Figure 1:**
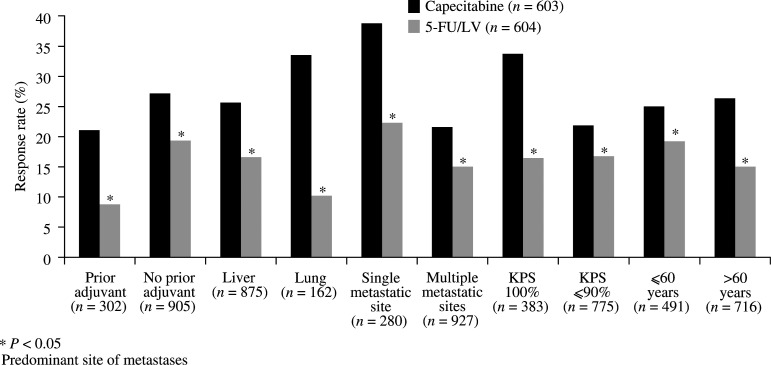
Response rates by subpopulation.

PR=partial response, CR=complete response.

). Notably, the superior efficacy of capecitabine was confirmed by the IRC-assessed response rate (22 *vs* 13%, *P*<0.0001). Furthermore, analysis of the data according to subpopulations defined by baseline characteristics consistently demonstrated superior response rates for capecitabine compared with 5-FU/LV (*P*<0.05; Figure 1

). In both treatment arms, response rates decreased with increasing numbers of metastatic sites, as would be expected. Of note, in patients who had previously received adjuvant chemotherapy, the response rate with capecitabine was 21% (31 out of 147 patients) compared with only 9% (14 out of 155) in patients treated with 5-FU/LV.

Response to treatment occurred at least as rapidly in patients treated with capecitabine compared with patients who received 5-FU/LV (median time to response: 1.7 *vs* 2.4 months, respectively). The median duration of response was also similar in the two treatment groups (8.1 and 9.4 months in the capecitabine and 5-FU/LV groups, respectively).

### Time to disease progression

**Figure 2:**
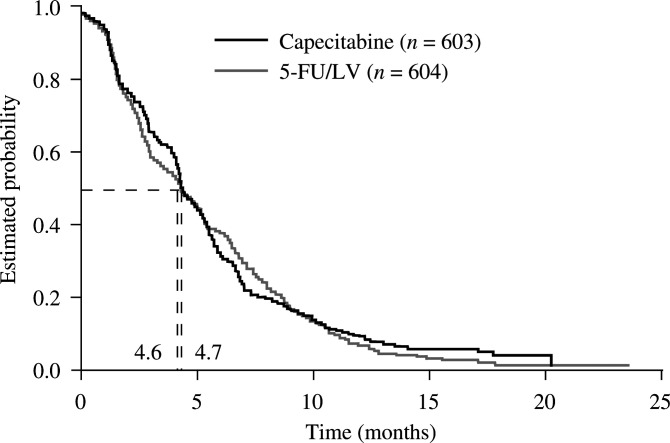
Time to disease progression.

TTP was equivalent in the two treatment groups (hazard ratio (HR) 0.997, 95% confidence interval (CI) 0.885−1.123, log-rank *P*=0.95). The median TTP was 4.6 months (95% CI 4.3−5.3) with capecitabine and 4.7 months (95% CI 4.3−5.4) with 5-FU/LV (Figure 2

). Subgroup analysis according to baseline characteristics, including cancer type (rectal *vs* colon cancer), history of adjuvant chemotherapy and gender, demonstrated no significant differences between the two treatment groups for TTP.

Regression analyses were undertaken to assess the impact of prognostic factors on TTP for the entire population. Univariate Cox regression analyses including treatment as a covariable were performed, and identified a number of factors associated with reduced TTP. These included poor Karnofsky performance status (KPS; 70 *vs* 100%), liver metastases, multiple metastatic sites and younger age, with prognostic significance at a level of 15% (Table 4

**Table 4:** Results of univariate Cox regression analysis: TTP

	**Both treatment groups**		**Capecitabine group**	
**Factor**	**HR**	***P*-value**	**HR**	***P*-value**
Liver metastases (yes *vs* no)	1.37	0.0001	1.39	0.0016
Metastatic sites (>1 *vs* 1)	1.39	0.0001	1.55	0.0001
*KPS*				
70 *vs* 100%	1.67	0.0009	1.61	0.0010
80 *vs* 100%	1.14	0.29	1.36	0.017
90 *vs* 100%	1.02	0.83	1.16	0.17
				
Previous adjuvant therapy (yes *vs* no)	1.00	0.96	0.92	0.39
Large (>10 patients) *vs* small (⩽10 patients) recruitment/treatment center	1.01	0.94	0.88	0.15
Age	0.99	0.045	0.99	0.0008
Gender	0.92	0.36	1.03	0.70

). Previous adjuvant treatment and gender did not appear to impact on disease progression. A multivariate Cox regression analysis using backward elimination of factors, with the first step including all factors identified in the univariate analyses, was then performed to assess the impact of independent prognostic factors on the treatment effect at a significance level of 5%. This analysis confirmed an increased risk of progression with lower age, liver metastases, poor KPS (70 or 80% at baseline) and multiple metastatic sites (Table 5

**Table 5:** Results of multivariate Cox regression analysis: TTP

	**Both treatment groups**		**Capecitabine group**	
**Factor**	**HR**	***P*-value**	**HR**	***P*-value**
Predominant metastatic site: liver	1.38	0.0001	1.46	0.0001
Metastatic sites (>1 *vs* 1)	1.42	0.0001	1.61	0.0001
*KPS*				
70 *vs* 100%	1.55	0.0001	1.52	0.0018
80 *vs* 100%	NA	NA	1.32	0.018

Age	0.99	0.013	0.99	0.0001

NA=not available.

). Of note, the analyses confirmed that risk of disease progression was independent of treatment, as indicated by the HR of 1.02 (95% CI 0.90–1.15; *P*=0.79).

### Overall survival

**Figure 3:**
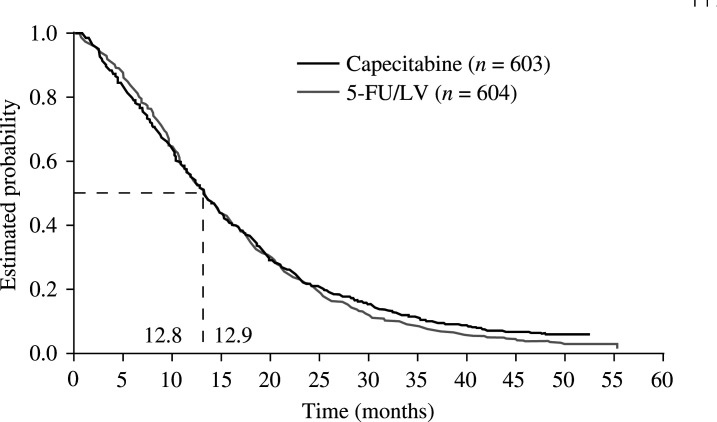
Overall survival: updated June 2002.

Overall survival data, updated in June 2002 after 1147 events, confirm the integrated analysis results reported by Twelves (2002). In patients receiving capecitabine, overall survival was equivalent to that in patients treated with 5-FU/LV (HR 0.95, 95% CI 0.84−1.06, *P*=0.48). The median survival was 12.9 months (95% CI 11.8−14.0) in the capecitabine group and 12.8 months (95% CI 11.7−14.0) in the 5-FU/LV group after 583 and 564 events, respectively (Figure 3

). Univariate analyses identified nine prognostic factors for survival with a significance level of 15%. Multivariate Cox regression analysis confirmed that lower KPS (70 or 80 *vs* 100%), multiple metastatic sites and the presence of liver metastases were independent prognostic indicators for poor survival (Table 6

**Table 6:** Results of multivariate Cox regression analysis: survival

**Factor**	**HR**	***P*-value**
Treatment	0.99 (0.87–1.13)	0.90
Country Mexico	2.71	0.0002
Predominant metastatic site: liver	1.38	0.0001
*KPS*		
70 *vs* 100%	2.34	0.0001
80 *vs* 100%	1.66	0.0001

Differentiation (well *vs* other)	0.77	0.016
Previous surgery	0.77	0.014
Number of metastatic sites	1.06	0.013
Metastatic sites (1 *vs* more)	1.29	0.013
Race ‘other’^a^	0.64	0.033

a Oriental (*n*=8), Hispanic (*n*=23) and all races other than Caucasian and Black.

). Patients with well-differentiated tumours had a slightly reduced risk of death compared with patients having less well-differentiated tumours. The data also suggest that previous surgery may have a prognostic effect, but the number of patients who did not have prior surgery was extremely small, as approximately 90% of patients had received prior surgery. Similarly, multivariate Cox regression analysis suggested that risk of death was markedly reduced in patients defined as race ‘other’ compared with the rest of the population (HR=0.64). However, this subgroup, comprising multiple ethnic groups (including Orientals and Hispanics), included only 35 patients in the capecitabine arm and 34 patients in the 5-FU/LV arm, and thus the results are difficult to interpret. Of note, the analyses confirmed that survival was independent of treatment. In addition to the predefined prognostic factors, baseline serum alkaline phosphatase, ASAT, serum albumin and haemoglobin were all confirmed as prognostic factors for survival in a secondary analysis (Table 7

**Table 7:** Prognostic value of baseline factors: survival

		**Wald test**
	**HR**	***P*-value**
*Alkaline phosphatase(U/l)*^a^	1.003	0.0001
Treatment interaction	1.000	0.91

*ASAT (IU)*	1.017	0.0001
Treatment interaction	1.003	0.33

Albumin (g dl)	0.975	0.0001
Treatment interaction	0.958	0.0001

*Haemoglobin (g dl)*	0.870	0.0001
Treatment interaction	0.971	0.39

a Normalised serum alkaline phosphatase.

).

Both irinotecan monotherapy (Cunningham *et al*, 1998; Rougier *et al*, 1998) and oxaliplatin in combination with 5-FU/LV (André *et al*, 1999; Maindrault-Goebel *et al*, 1999) have proven efficacy in patients with mCRC that has progressed during/following previous 5-FU-based therapy. Therefore, the impact of second-line chemotherapy on survival in the capecitabine phase III trials was also evaluated. The results of this analysis demonstrated that a similar number of patients in the capecitabine and 5-FU/LV groups received second-line chemotherapy (55 and 56% of patients, respectively). However, the choice of treatment was different for patients in the two treatment groups: among patients receiving second-line therapy, those in the capecitabine group received second-line 5-FU-based treatment more frequently than patients in the 5-FU/LV arm (54 *vs* 35%, *P*=0.00001, Table 8

**Table 8:** Second-line chemotherapy: incidence, timing and duration

	**Capecitabine**			**5-FU/LV**		
**Second-line treatment**	**% of patients receiving second-line therapy (*n*=334)**	**Treatment start^a^**	**Duration (days)**	**% of patients receiving second-line therapy (*n*=340)**	**Treatment start^a^**	**Duration (days)**
Irinotecan	29	192	66	41	185	60
Oxaliplatin	3	289	71	6	169	71
5-FU	54	168	59	35	194	64

a Median days from randomisation to start of second-line chemotherapy.

) (Twelves, 2001). Consequently, second-line irinotecan therapy was administered to a smaller proportion of those patients who received second-line treatment in the capecitabine arm than in the 5-FU/LV arm (29 *vs* 41%, respectively, *P*<0.001) (Twelves, 2001). The timing and duration of second-line treatment was similar in the two groups.

## DISCUSSION

The common mode of action of 5-FU and oral fluoropyrimidines predicts similar efficacy for these two approaches. For this reason, trials comparing oral fluoropyrimidines and conventional i.v. 5-FU/LV regimens in the treatment of colorectal cancer generally require demonstration of equivalent efficacy between the two agents. However, the results of this integrated analysis demonstrated that as first-line therapy for mCRC, capecitabine offers a superior response rate and equivalent time to progression and survival compared with i.v. bolus 5-FU/LV. The efficacy of capecitabine therefore compares favourably with that of other oral fluoropyrimidines evaluated in patients with mCRC. Recently, two large, independent, phase III trials in a total of more than 1400 patients demonstrated that the dihydropyrimidine dehydrogenase inhibitor eniluracil in combination with oral 5-FU is less effective than 5-FU/LV (Mayo Clinic regimen) as first-line treatment for mCRC (Van Cutsem *et al*, 2001b; Schilsky *et al*, 2002). Overall survival was significantly inferior with eniluracil/oral 5-FU *vs* 5-FU/LV in one trial and TTP was significantly inferior with eniluracil/5-FU in the other. Similarly, a large phase III trial in 816 patients failed to demonstrate equivalent efficacy for UFT/LV and 5-FU/LV (Mayo Clinic regimen). In this study, the risk of disease progression was increased by 22% in patients receiving the investigational therapy, UFT/LV (*P*=0.011, HR 0.823, 95% CI 0.708–0.958) (Douillard *et al*, 2002; Schmoll, 2003). Whereas the current analysis demonstrates superior response rates for capecitabine *vs* 5-FU/LV, the overall response rates for UFT/LV and 5-FU/LV did not differ significantly between treatment arms, with a trend to a lower response rate for UFT/LV. Capecitabine is the only oral fluoropyrimidine that has demonstrated efficacy at least equivalent to that of 5-FU/LV (Mayo Clinic regimen) as first-line therapy for colorectal cancer, leading to its regulatory approval worldwide in this indication.

The superior response rate observed with capecitabine compared with 5-FU/LV was seen consistently in all subpopulation analyses. Patients with poor prognostic indicators, such as poor KPS and liver metastases, were more likely to respond if treated with capecitabine than with 5-FU/LV. The superior response rate was particularly pronounced in the subpopulation of patients who had previously received adjuvant 5-FU. However, even among patients not previously exposed to fluoropyrimidines of any kind, the response rate was superior for those receiving capecitabine.

A multivariate analysis of survival to evaluate the impact of prognostic factors confirmed the primary analysis of survival, demonstrating that survival was independent of treatment administered. Evaluation of the impact of second-line treatment on survival outcomes demonstrated that poststudy treatment favoured the 5-FU/LV group, and therefore strengthens the claim that capecitabine results in efficacy at least equivalent to that achieved with 5-FU/LV.

An integrated analysis of safety data from the phase III trials has also demonstrated that capecitabine has an improved safety profile compared with 5-FU/LV, with a significantly (*P*<0.001) lower incidence of diarrhoea (47.7 *vs* 58.2%), stomatitis (24.3 *vs* 61.6%), nausea (37.9 *vs* 47.6%) and alopecia (6.0 *vs* 20.6%) (Cassidy *et al*, 2002). The only adverse event occurring significantly more frequently with capecitabine was hand-foot syndrome (53.5 *vs* 6.2% with 5-FU/LV, *P*<0.001). This cutaneous side effect is readily managed by treatment interruption and dose reduction and led to hospitalisation of only two patients (both for <24 h). The lower incidence of Grade 3–4 neutropaenia (2.3 *vs* 22.8%) with capecitabine compared with 5-FU/LV led to significantly (*P*<0.001) less neutropaenic fever/sepsis (0.2 *vs* 3.4%) and consequently fewer hospitalisations (Cassidy *et al*, 2002). Grade 4 adverse events were more common with 5-FU/LV than with capecitabine (5.1 *vs* 3.0%, respectively; *P*=0.078), mostly comprising neutropaenia-related complications and diarrhoea. The incidence of grade 3 or 4 treatment-related adverse events during the first treatment cycle was significantly higher in patients receiving 5-FU/LV than in those receiving capecitabine (22.6 *vs* 9.1%, respectively; *P*<0.001).

The results of this integrated analysis therefore support the use of capecitabine as first-line monotherapy for advanced colorectal cancer. As an effective oral agent, capecitabine meets patients' needs for a convenient, oral treatment suitable for outpatient therapy. In addition, analysis of medical resource use demonstrated that significantly fewer patients required hospitalisation for treatment-related adverse events (11.6 *vs* 18.0%, *P*<0.005), and fewer physician visits were required for treatment administration with capecitabine than with 5-FU/LV (4 *vs* 15 in a 12-week period).

Combination chemotherapy is becoming increasingly common in patients who can tolerate intensive therapy. Two phase III studies have demonstrated that the addition of irinotecan to bolus or infused 5-FU/LV provides a modest but statistically significant survival benefit in the first-line treatment of patients with colorectal cancer (Douillard *et al*, 2000; Saltz *et al*, 2000). However, there are certain patient subgroups for whom first-line combination therapy may not be the most appropriate treatment strategy. For example, in patients with poor performance status and elevated serum LDH, irinotecan/5-FU/LV combination therapy did not appear to confer a survival benefit. In the subgroup of patients who had previously received adjuvant therapy, overall survival was reduced compared with the overall patient population (FDA Medical Officer Summary, 1999; Knight *et al*, 2000). In a multivariate analysis of almost 4000 patients, Köhne *et al* (2002) identified four clinical parameters (performance status, WBC count, alkaline phosphatase concentration and number of involved tumour sites) enabling grouping of patients into low-, medium- or high-risk categories. Assessment of risk for each patient potentially facilitates decisions on whether more or less intensive treatments are most appropriate for each individual. Recently published National Comprehensive Cancer Network (NCCN) guidelines (National Comprehensive Cancer Network Clinical Practice Guidelines in Oncology, 2003) recommend that in patients who cannot tolerate intensive combination therapy, fluoropyrimidine monotherapy is the most appropriate treatment strategy. In this context, capecitabine provides a highly active first-line treatment option. The ECOG and EORTC are currently planning a study in poor-prognosis patients comparing capecitabine monotherapy *vs* capecitabine/irinotecan combination therapy *vs* capecitabine/oxaliplatin combination therapy. Results of this trial should provide insight into the optimisation of treatment strategies for patients with a poor prognosis.

Another important consideration when comparing sequential *vs* combination therapy in the first-line setting is the tolerability of the two approaches. The analyses of the NCCTG 9741 and CALGB 89803 randomised phase III trials have raised concerns about the safety of irinotecan in combination with bolus 5-FU/LV, with an unexpectedly high number of early deaths leading to modification or closure of treatment arms combining bolus 5-FU/LV regimens with irinotecan or oxaliplatin (Morton *et al*, 2001; Rothenberg *et al*, 2001; Ratain, 2002).

Irinotecan/fluoropyrimidine therapy might be better tolerated when 5-FU/LV is administered as a protracted infusion, and recent data from the NCCTG trial also suggest that combination therapy with infused 5-FU/LV may provide efficacy advantages over those incorporating a bolus regimen (Goldberg *et al*, 2004). Capecitabine, which approximates to continuous infusion 5-FU and has an improved safety profile compared with bolus 5-FU, potentially provides a better-tolerated combination partner for irinotecan. Recently results from two trials evaluating first-line capecitabine plus irinotecan, (*n*=37 and 52) showed response rates of 43 and 46% and median time to progression of 9.3 and 7.1 months, respectively (Borner *et al*, 2003; Patt *et al*, 2003). Phase III evaluation of capecitabine/irinotecan combination therapy is ongoing. Capecitabine is also in the early stages of evaluation in combination with oral irinotecan, thus offering potential for all-oral combination therapy for patients with colorectal cancer.

Similarly, clinical studies have demonstrated that oxaliplatin in combination with infused 5-FU/LV is a highly effective first-line treatment for patients with advanced colorectal cancer, resulting in superior response rates and TTP compared with 5-FU/LV alone (de Gramont *et al*, 2000; Giacchetti *et al*, 2000). In preclinical models the combination is more effective if 5-FU is administered as a continuous infusion. This observation suggests that replacing cumbersome i.v. 5-FU infusions with oral capecitabine may represent a more effective, more convenient oxaliplatin combination therapy than current i.v. 5-FU-based regimens. Mature results of a phase II, multicentre trial of capecitabine plus oxaliplatin in 96 patients has demonstrated an overall response rate of 55%, with consistently high (>50%) response rates across all patient subgroups studied (Van Cutsem *et al*, 2003). In this trial the median progression-free survival was 7.7 months and median overall survival was 19.5 months. The regimen had a favourable safety profile, with a low incidence of grade 3 or 4 treatment-related adverse events.

An extensive phase III programme is evaluating both capecitabine plus irinotecan and capecitabine plus oxaliplatin with or without biological agents (bevacizumab) in first-line and in the adjuvant setting.

In addition to combination with irinotecan and oxaliplatin for the treatment of advanced colorectal cancer, capecitabine has been evaluated as a combination partner for radiotherapy in the management of rectal cancer. Preclinical studies demonstrated that capecitabine and radiotherapy have enhanced antitumour activity, which is most likely attributable to the further upregulation of thymidine phosphorylase (the enzyme responsible for the final conversion of capecitabine to 5-FU) in tumour cells following radiotherapy (Sawada *et al*, 1999). More recently, a phase II study demonstrated that capecitabine/radiotherapy combination treatment is feasible with promising activity (Dunst *et al*, 2003). Phase II evaluation, particularly in the neo-adjuvant setting, is ongoing and a phase III trial (NSABP R-04) will compare chemoradiation with capecitabine *vs* protracted infusion 5-FU. The addition of oxaliplatin or irinotecan to capecitabine could further improve chemoradiation efficacy outcomes in the future, and phase I trials are ongoing.

Capecitabine is also an attractive agent for use in the adjuvant setting. A phase III trial to evaluate capecitabine treatment for Dukes' C colon cancer completed patient accrual in 2001 and the safety and efficacy results are eagerly awaited.

The results of this integrated efficacy analysis have confirmed the results of the two individual trials, showing that capecitabine is a highly effective agent for the first-line treatment of advanced colorectal cancer. As first-line therapy, capecitabine results in superior response rate, is more convenient and has an improved safety profile compared with 5-FU/LV. Phase I and II studies evaluating capecitabine in combination regimens indicate that capecitabine is a very promising and suitable candidate to replace 5-FU as the backbone of colorectal cancer chemotherapy. Phase III trials should elucidate whether capecitabine may become the backbone of colorectal cancer combination therapy, not only with irinotecan, oxaliplatin and radiotherapy but also with novel agents such as EGFR inhibitors and anti-angiogenic agents.
